# Hexavalent-Chromium-Induced Disruption of Mitochondrial Dynamics and Apoptosis in the Liver via the AMPK-PGC-1α Pathway in Ducks

**DOI:** 10.3390/ijms242417241

**Published:** 2023-12-08

**Authors:** Chang Wang, Xueyan Dai, Chenghong Xing, Caiying Zhang, Huabin Cao, Xiaoquan Guo, Ping Liu, Fan Yang, Yu Zhuang, Guoliang Hu

**Affiliations:** Jiangxi Provincial Key Laboratory for Animal Health, Institute of Animal Population Health, College of Animal Science and Technology, Jiangxi Agricultural University, No. 1101 Zhimin Avenue, Nanchang 330045, China

**Keywords:** hexavalent chromium, AMPK-PGC-1α pathway, mitochondrial dynamics, apoptosis, liver

## Abstract

Hexavalent chromium (Cr(VI)) is a hazardous substance that poses significant risks to environmental ecosystems and animal organisms. However, the specific consequences of Cr(VI) exposure in terms of liver damage remain incompletely understood. This study aims to elucidate the mechanism by which Cr(VI) disrupts mitochondrial dynamics, leading to hepatic injury in ducks. Forty-eight healthy 8-day-old ducks were divided into four groups and subjected to diets containing varying doses of Cr(VI) (0, 9.28, 46.4, and 232 mg/kg) for 49 days. Our results demonstrated that Cr(VI) exposure resulted in disarranged liver lobular vacuolation, along with increasing the serum levels of ALT, AST, and AKP in a dose-dependent manner, which indicated liver damage. Furthermore, Cr(VI) exposure induced oxidative stress by reducing the activities of T-SOD, SOD, GSH-Px, GSH, and CAT, while increasing the contents of MDA and H_2_O_2_. Moreover, Cr(VI) exposure downregulated the activities of CS and MDH, resulting in energy disturbance, as evidenced by the reduced AMPK/p-AMPK ratio and PGC-1α protein expression. Additionally, Cr(VI) exposure disrupted mitochondrial dynamics through decreased expression of OPA1, Mfn1, and Mfn2 and increased expression of Drp-1, Fis1, and MFF proteins. This disruption ultimately triggered mitochondria-mediated apoptosis, as evidenced by elevated levels of caspase-3, Cyt C, and Bax, along with decreased expression of Bcl-2 and the Bcl-2/Bax ratio, at both the protein and mRNA levels. In summary, this study highlights that Cr(VI) exposure induces oxidative stress, inhibits the AMPK-PGC-1α pathway, disrupts mitochondrial dynamics, and triggers liver cell apoptosis in ducks.

## 1. Introduction

Chromium (Cr), the 20th most common trace element, is typically obtained from chromate ore (FeCr_2_O_4_) found in nature. Cr exists in various oxidation states, including Cr^1+^, Cr^2+^, Cr^3+^, Cr^4+^, Cr^5+^, and Cr^6+^; among these states, Cr^6+^ is particularly stable [1,2]. Within the Agency for Toxic Substances and Disease Registry (ATSDR), Cr(VI) ranks seventh in terms of toxicity and is regarded as highly toxic and as one of the most potent carcinogens [3]. The primary occupational sources of environmental Cr(VI) pollution include leather tanning, pigment and rubber production, paint manufacturing, anticorrosion agent production, and coal processing. Furthermore, occupational exposure can occur through the consumption of chromium-contaminated water and food, leading to significant detrimental effects on human health [4]. Recent studies have demonstrated that exposure to Cr(VI) can result in irreversible organ damage, such as apoptosis and necrosis, through inhalation, ingestion, or skin contact [5]. Liver, lung, and kidney damage are especially prominent among the toxic effects induced by Cr(VI), raising considerable concerns regarding biosafety [6].

Accumulating evidence has revealed that Cr(VI) can be attributed to its ability to generate reactive oxygen species (ROS) through the Fenton reaction, including nitric oxide, superoxide anions, hydroxyl radicals, and hydrogen peroxide [7]. The disruption of the oxidant/antioxidant system equilibrium by Cr(VI) induces oxidative stress, resulting in detrimental consequences such as protein damage, DNA fragmentation, lipid peroxidation, and gene expression alteration, ultimately leading to cellular damage and apoptosis [8,9]. The liver serves as both the primary metabolic organ and a site for accumulating various toxic substances. Continuous exposure to chromium trioxide can cause human liver damage, including liver cell malfunction, necrosis, and inflammatory cell infiltration [10]. Cr(VI) exposure caused swelling of mitochondria, breakage of mitochondrial cristae, and loss of mitochondrial cristae with increasing dose in rat livers [11]. Additionally, previous research has demonstrated the significance of apoptosis and oxidative stress in Cr(VI)-induced liver injury [12]. A study on Cr(VI) exposure showed that it caused apoptosis of hepatocytes and elevated levels of Bax and caspase-3 in broiler chickens [13].

Mitochondria, essential for energy production and cellular homeostasis in eukaryotic cells, possess a highly dynamic double-membrane-bound structure. This plays a critical role in various cellular processes, including oxidative phosphorylation (OXPHOS)-mediated energy production, apoptosis, calcium homeostasis, and multiple biological functions [14]. Mitochondrial dynamics, characterized by the continuous fission and fusion of mitochondria, orchestrates the number, size, and positioning of mitochondria to generate a coordinated tubular network [15,16]. A variety of evidence has demonstrated that mitochondrial dynamic processes are vital for maintaining the normal functionality of mitochondria. However, in the presence of detrimental conditions like exposure to toxicants and heavy metals, mitochondrial fission and fusion become disrupted, thereby impairing energy homeostasis and leading to mitochondrial damage [17,18]. Previous studies have revealed that Cr(VI) induces structural impairment in testicular cell mitochondria and decreases ATP levels in a dose-dependent manner. Furthermore, Cr(VI) was found to interfere with the expression of key genes involved in mitochondrial dynamics, namely, Drp-1 and Mfn2, thereby promoting mitochondrial division and inhibiting mitochondrial fusion [13,19].

AMP-activated protein kinase (AMPK) and peroxisome proliferator-activated receptor gamma coactivator 1-alpha (PGC-1α) exert critical regulatory control over energy metabolism and mitochondrial dynamics [20,21,22]. Activation of AMPK leads to upregulation of PGC-1α expression, a transcriptional coactivator essential for mitochondrial biogenesis and energy metabolism. Additionally, AMPK phosphorylates PGC-1α, augmenting its transcriptional activity and exerting further influence on mitochondrial function and energy metabolism [23]. This intricate interplay between AMPK and PGC-1α provides a sophisticated regulatory mechanism governing energy expenditure and metabolic adaptation, holding promise for therapeutic interventions targeting mitochondrial dysfunction and metabolic disorders [24]. Previous studies have demonstrated that exposure to Cr(VI) leads to elevated levels of ROS, causing severe oxidative stress in the livers of mice, while concurrently increasing AMPK phosphorylation to activate the apoptotic signaling pathway. Furthermore, following exposure to Cr(VI), higher levels of phosphorylation were observed in protein kinase B (AKT) and extracellular regulated kinase (ERK), leading to mitochondrial dysfunction and activation of the caspase pathway, thereby inducing cell damage [25,26,27].

The duck *Anas platyrhyncha*, a breed predominantly found in Southern China and a major breed in the Cr(VI)-polluted region of southern Jiangxi Province, provides a suitable model for studying the toxic effects of Cr(VI). Several studies have elucidated the mechanisms of organ damage induced by Cr(VI). However, the precise toxic effects of Cr(VI) on liver damage remain largely unknown. Therefore, this study focuses on investigating the molecular mechanisms of liver injury caused by Cr(VI) by examining the relationships among oxidative stress, mitochondrial fission and fusion, and apoptosis.

## 2. Results

### 2.1. Cr(VI) Induced Structural and Pathological Damage in Duck Livers

**Figure 1:**
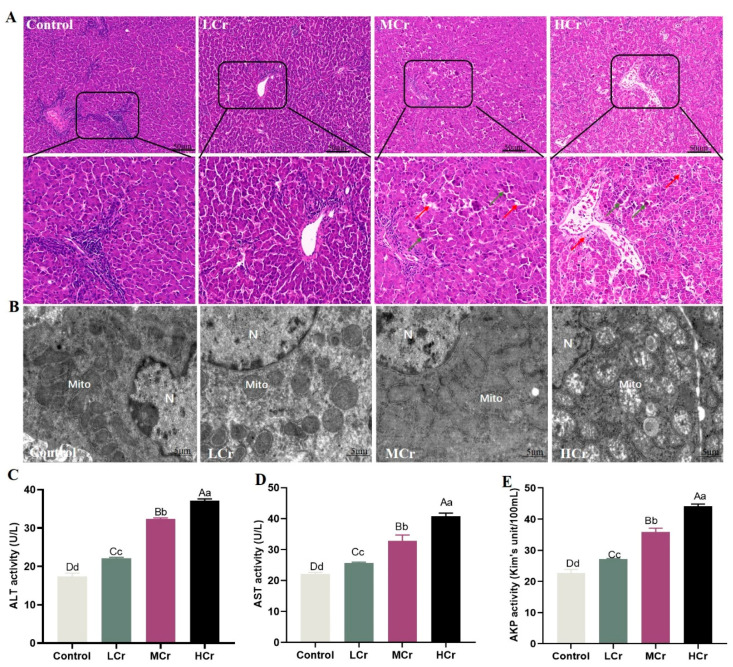
Cr(VI)-induced histopathological damage and dysfunction in the liver: (**A**) The histological features (magnification: ×200 and ×400, scale bar = 50 μm). The white arrow represents interstitial hepatic cords, the red arrow represents cellular steatosis, and the green arrow represents inflammatory cells. (**B**) Ultrastructural features (magnification: ×1200, scale bar = 5 μm). N: nucleus; Mito: mitochondria. (**C**) ALT activity. (**D**) AST activity. (**E**) AKP activity. Note: bars represent the mean ± standard deviation. With the column data, the same letters on the shoulder mean that the difference is not significant (*p* > 0.05), different small letters mean a significant difference (*p* < 0.05), and different capital letters mean an extremely significant difference (*p* < 0.01).

In order to research the effects of Cr(VI) on the duck liver, we estimated the degree of pathological damage in each group at 49 days. As shown in Figure 1A, compared with the control group, H&E staining revealed that the livers had a compromised structure, the hepatic sinusoidal space shrank, and the radial hepatic cord vanished in the Cr(VI)-treated group. Meanwhile, Cr(VI) exposure caused liver cell swelling, necrotic fatty degeneration, and increased the number of inflammatory cells. Notably, these changes had a dose-dependent effect on liver cell damage.

### 2.2. Cr(VI) Induced Mitochondrial Ultrastructural Changes in the Liver

The TEM results showed that the structure of the mitochondria was complete and clear in the control group. Conversely, the ridges of the mitochondria were slightly fractured and somewhat enlarged in the LCr group. The mitochondrial morphology was muddled and the mitochondria seemed inflated and dilated in the MCr group. In the HCr group, there significant changes to the mitochondrial structure, including enlargement, rupture, disappearing ridges, and even vacuolated lesions (Figure 1B).

### 2.3. Cr(VI) Increased Serum Liver Function Indices to Induce Liver Injury

The results of the liver function indicators are shown in Figure 1C–E. The activities of AST, ALT, and AKP in the serum significantly rose with increasing Cr(VI) dosages in all Cr(VI)-treated animals, indicating that Cr(VI) can harm the liver.

### 2.4. Cr(VI) Destroyed the Antioxidant Balance to Induce Oxidative Stress in the Liver

**Figure 2:**
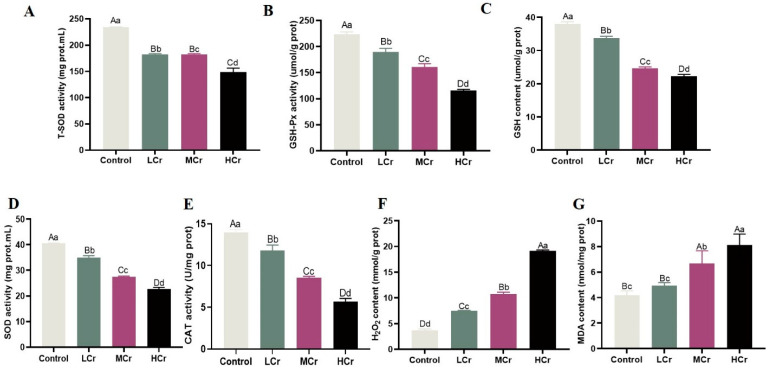
Cr(VI) destroyed the antioxidant balance to induce oxidative stress in the liver: (**A**) T-SOD activity; (**B**) GSH-Px activity; (**C**) GSH content; (**D**) SOD activity; (**E**) CAT activity; (**F**) H_2_O_2_ content; (**G**) MDA content. With the column data, the same letters on the shoulder mean that the difference is not significant (*p* > 0.05), different small letters mean a significant difference (*p* < 0.05), and different capital letters mean an extremely significant difference (*p* < 0.01).

By evaluating T-SOD, SOD, GSH-Px, and CAT activities along with MDA, GSH, and H_2_O_2_ levels in the liver tissue of ducks exposed to Cr(VI), we determined whether the antioxidant system was in equilibrium or not. According to Figure 2A–E, the T-SOD, SOD, GSH-Px, GSH, and CAT activities were significantly reduced and dose-dependent in the Cr(VI)-treated groups. Additionally, the H_2_O_2_ and MDA levels were obviously increased and dose-dependent (Figure 2F,G).

### 2.5. Cr(VI) Interfered with the Energy Balance through Inhibiting the AMPK/PGC-1α Signaling Pathway in the Liver

**Figure 3:**
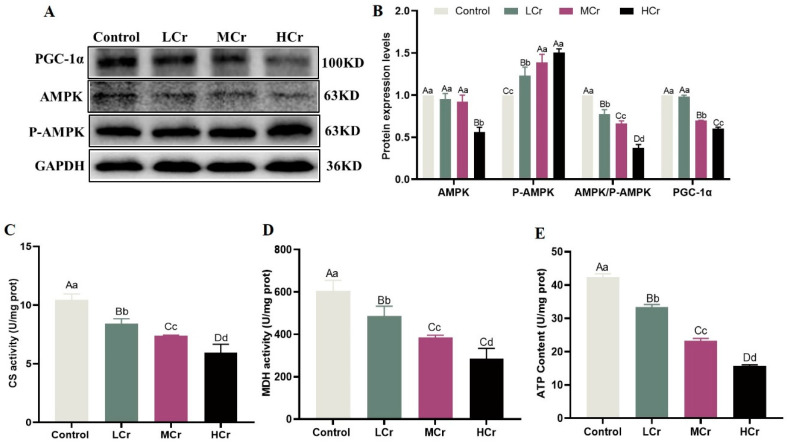
Cr(VI) interfered with the energy balance through inhibiting the AMPK/PGC-1α signaling pathway in the liver (**A**) AMPK and PGC-1α protein bands; (**B**) AMPK and PGC-1α protein expression levels; (**C**) CS activity; (**D**) MDH activity; (**E**) ATP content. With the column data, the same letters on the shoulder mean that the difference is not significant (*p* > 0.05), different small letters mean a significant difference (*p* < 0.05), and different capital letters mean an extremely significant difference (*p* < 0.01).

As can be seen in Figure 3A,B, there was a dose-dependent downregulation of AMPK and PGC-1α expression in the Cr(VI) treatment groups compared to the control group, but there was also a sharp decline in the AMPK/p-AMPK ratio. The results demonstrated that Cr(VI) treatment significantly reduced the intracellular ATP content and the activities of CS and MDH, and this impact was also related to the dose of Cr(VI) exposure (Figure 3C–E).

### 2.6. Cr(VI) Inhibited Drp1-Mediated Mitochondrial Fusion and Promoted Fis1-Mediated Mitochondrial Fission in the Liver

**Figure 4:**
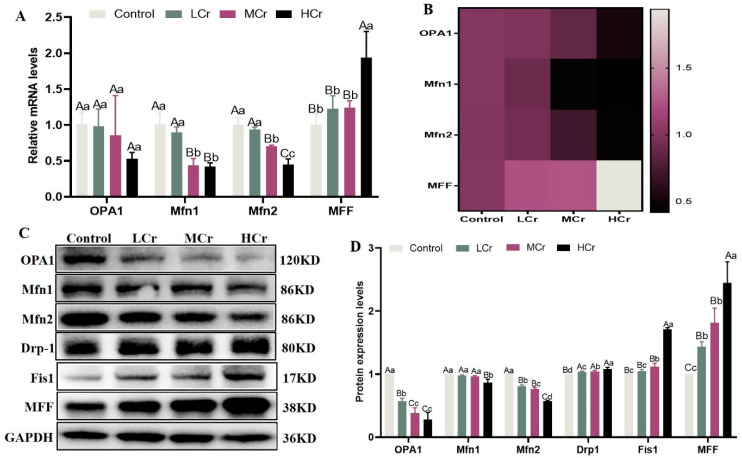
Cr(VI) inhibited Drp1-mediated mitochondrial fusion and promoted Fis1-mediated mitochondrial fission in the liver: (**A**) Relative mRNA expression levels of mitochondrial dynamics. (**B**) Heatmap analysis of mitochondrial-dynamics-related genes’ mRNA expression levels. (**C**) Protein bands. (**D**) Relative protein expression levels of mitochondrial dynamics. With the column data, the same letters on the shoulder mean that the difference is not significant (*p* > 0.05), different small letters mean a significant difference (*p* < 0.05), and different capital letters mean an extremely significant difference (*p* < 0.01).

The results from RT-qPCR revealed a downward trend in the mRNA expression of *OPA1*, *Mfn1*, and *Mfn2*, while the mRNA expression of *MFF* exhibited an upward trend relative to the control group (Figure 4A,B). Furthermore, Western blot analysis showed that the livers from the Cr(VI)-exposed groups had increased translation levels of the fission proteins Drp-1, Fis1, and MFF. In contrast, the fusion proteins Mfn1, Mfn2, and OPA1 were translated at lower levels. These were closely related to the doses of Cr(VI) exposure (Figure 4C,D).

### 2.7. Cr(VI) Promoted the Apoptosis of Mitochondrial Pathways in the Liver

**Figure 5:**
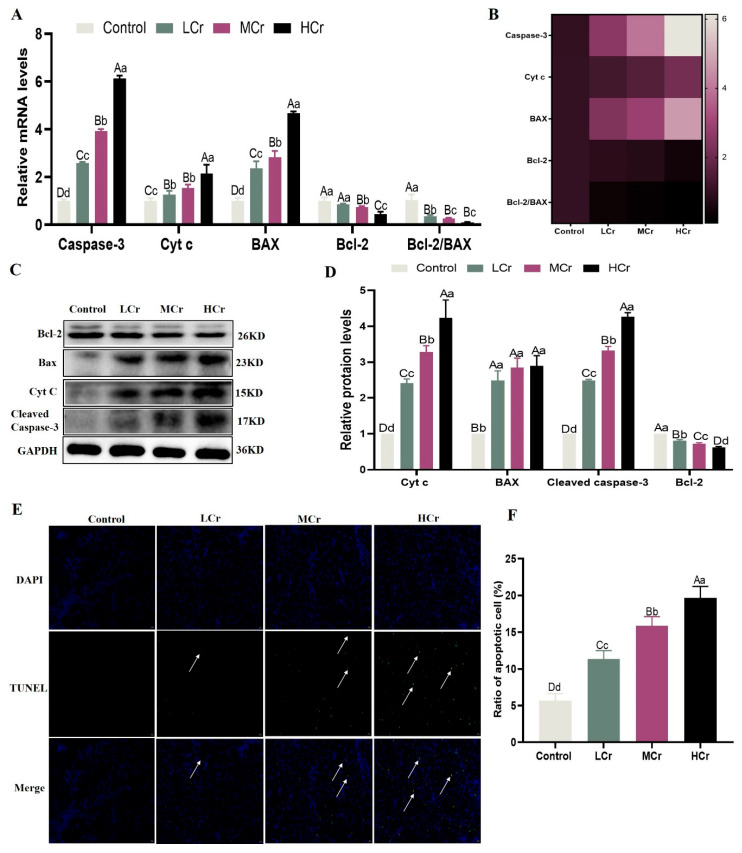
Cr(VI) promoted the apoptosis of mitochondrial pathways in the liver: (**A**) Relative mRNA expression levels of apoptosis. (**B**) Heatmap analysis of apoptosis related genes’ mRNA expression levels. (**C**) Protein bands. (**D**) Relative protein expression levels of apoptosis. (**E**) Representative images of TUNEL-positive hepatocytes (400×); bars = 20 μm; white arrows: positive results. (**F**) Hepatocyte apoptosis rate analysis. With the column data, the same letters on the shoulder mean that the difference is not significant (*p* > 0.05), different small letters mean a significant difference (*p* < 0.05), and different capital letters mean an extremely significant difference (*p* < 0.01).

The RT-qPCR results demonstrated that the *caspase-3*, *Bax*, and *Cyt C* mRNA transcript levels were dramatically elevated, whereas the *Bcl-2* mRNA transcription level and the ratio of *Bcl-2/Bax* were greatly decreased, in all Cr(VI) treatment groups (Figure 5A,B). As shown in Figure 5C,D, the expression levels of their proteins were consistent with genetic levels. Additionally, we evaluated the hepatocyte apoptosis level by TUNEL staining, as presented in Figure 5E,F. The few TUNEL-positive cells were found in the control group. On the other hand, with the increase in the Cr(VI) dose, the number of TUNEL-positive cells increased dramatically.

### 2.8. Correlation Analysis

**Figure 6:**
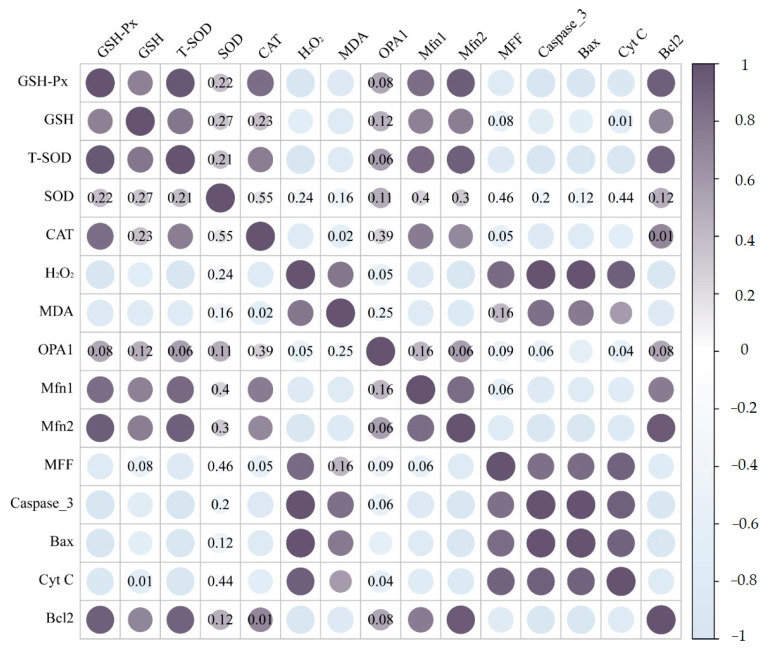
Correlation analysis among oxidative stress, mitochondrial dynamics, and apoptosis in the livers of ducks.

The relationships among oxidative stress, mitochondrial dynamics, and apoptosis were further verified by correlation analysis. As described in Figure 6, there was a strikingly positive association between MDA content, H_2_O_2_, and the expression levels of the mitochondrial fission gene (*MFF*) and apoptosis genes (*caspase-3*, *Bax*, *Cyt C*). The expression levels of oxidative stress (GSH-Px, GSH, T-SOD, and CAT) and mitochondrial fusion factors (*Mfn1*, *Mfn2*, and *OPA1*), on the other hand, appeared to be negatively correlated. 

## 3. Discussion

Cr(VI) is recognized as one of the most hazardous heavy metals, capable of inflicting detrimental effects on human health, including liver and kidney damage, internal hemorrhage, and respiratory disorders [28,29]. Additionally, it has been classified as a Group I human carcinogenic substance by the International Agency for Research on Cancer [30,31]. Among the affected organs, the liver is particularly vulnerable to the toxicity of Cr(VI). Previous investigations have demonstrated the ability of Cr(VI) to induce severe liver damage; serum levels of ALT and AST are massive biochemical indicators of liver injury, and exposure to Cr(VI) results in a reduction in cell membrane permeability as well as an increase in AST and ALT leakage [12]. The histopathological results and biochemical indices of this study suggested that Cr(VI) exposure caused damage to ducks’ livers. However, the underlying molecular mechanisms remain poorly understood. In this study, our findings revealed that exposure to Cr(VI) leads to oxidative stress, suppresses the activity of the AMPK-PGC-1α pathway, disrupts mitochondrial dynamics, and ultimately triggers liver cell damage and apoptosis.

Heavy metal toxicity can lead to oxidative stress, causing the accumulation of reactive oxygen species (ROS) and damage to cellular components. Among them, mitochondria are the primary organelles targeted by ROS and the primary site of ROS generation. Over-fission of the mitochondria lowers the antioxidant capacity and damages the respiratory chain and mitochondria, promoting the generation of ROS and allowing electron leakage of the respiratory chain complexes in the mitochondria [32,33]. MDA reflects lipid peroxidation and the severity of oxidative damage. SOD, T-SOD, and CAT are important antioxidants that protect against oxidative damage [34,35] GSH and GSH-Px help eliminate intracellular ROS and protect cell membranes. Previous studies have shown that Cr(VI) exposure increases H_2_O_2_ and MDA levels while decreasing T-SOD and CAT activities, leading to oxidative stress [34,36,37,38,39,40,41]. Xing et al. reported that exposure to Cr(VI) resulted in an increase in H_2_O_2_ and MDA levels, as well as a decrease in T-SOD and CAT activities, leading to oxidative stress in the intestinal barriers of ducks [42] Similarly, Cr(VI) has been shown to lower SOD and GSH levels while elevating MDA levels in the kidney tissue of broilers [43]. Consistent with prior studies, our findings indicate that sustained exposure to Cr(VI) attenuates the defense system against free radicals, characterized by a decline in T-SOD, SOD, GSH-Px, GSH, and CAT activities, coupled with elevated MDA and H_2_O_2_ levels. These findings indicate that prolonged Cr(VI) exposure induces oxidative stress in duck livers.

Mitochondrial dysfunction, triggered by oxidative stress, is a critical factor in disturbing cellular homeostasis. Accumulating evidence indicates that heavy metals possess the ability to significantly impact mitochondrial dynamics by perturbing the delicate balance between fusion and fission events, primarily driven by oxidative stress [44,45]. The regulation of mitochondrial dynamics involves various proteins, including Mfn1 and Mfn2 at the outer mitochondrial membrane (OMM), OPA1 at the inner mitochondrial membrane (IMM), and Drp1, Fis1, and MFF associated with fission [46]. The fusion process occurs distinctly at the OMM by Mfn1 and Mfn2, while the IMM’ fusion is coordinated by OPA1. On the other hand, mitochondrial division is facilitated by Drp1, which is recruited to the OMM through the OMM receptor Fis1, leading to constriction and scission. An imbalance in mitochondrial dynamics, characterized by the coexistence of fusion and fission proteins, represents a crucial event that triggers abnormal mitochondrial fragmentation. The present study demonstrated that exposure to Cr(VI) disrupts mitochondrial fusion via OPA1 and promotes enhanced mitochondrial fission mediated by Fis1, ultimately resulting in impaired mitochondrial fragmentation, in accordance with the ultrastructural observations of Cr(VI)-exposed mitochondria in the liver.

Mitochondria play a pivotal role in providing energy for vital cellular functions and exhibit adaptability to diverse metabolic conditions via AMPK pathways that activate PGC-1α, thereby maintaining mitochondrial homeostasis by regulating both mitochondrial dynamics and energetics. PGC-1α is essential in regulating mitochondrial dynamics and functionality through its primary interactions with Drp-1 and Mfn2. In line with these findings, the study by Xue et al. revealed that exposure to Cr(VI) disrupts mitochondrial dynamics by suppressing the AMPK-PGC-1α pathway, downregulating mitochondrial fusion genes, and upregulating mitochondrial fission genes [13]. Consistently, previous studies have observed a notable increase in Drp-1 expression and inhibition of Mfn2, leading to excessive mitochondrial fragmentation, specifically in the kidneys and testes of rats exposed to Cr(VI) [47,48]. Our findings suggest that the modulation of mitochondrial dynamics likely occurs through an AMPK-PGC-1α-mediated mechanism, leading to altered expression of mitochondrial fusion–fission proteins.

Dysregulation of mitochondrial fusion and fission processes profoundly compromises the overall mitochondrial functionality, leading to diminished energy output and potentially culminating in cell death [49,50]. In the presence of oxidative stress, crucial enzymes involved in the tricarboxylic acid (TCA) cycle, such as CS and malate dehydrogenase (MDH), are inhibited, resulting in impaired ATP production within the mitochondria [51]. Lai et al. reported a dose-dependent decrease in the activities of key enzymes involved in the TCA cycle, such as CS and MDH, upon exposure to manganese (Mn) [52]. Our results provide additional evidence for the inhibitory effects of Cr(VI) on mitochondrial fusion, promotion of mitochondrial division, disruption of mitochondrial dynamics, and impairment of ATP production, and the severity of mitochondrial fragmentation was also observed by TEM analysis. 

Accumulating evidence suggests a strong relationship between mitochondrial dynamics disorders and apoptosis. A recent study found that the anti-apoptotic protein myeloid cell leukemia 1 (MCL-1) caused mitochondrial fission and inhibited the mortality of cardiomyocytes, indicating that mitochondrial fission provides protection against stress-driven cell death [53]. However, some studies have also indicated that an imbalance in mitochondrial dynamics, such as excessive mitochondrial fission, induces mitochondria damage, eventually leading to mitochondria-dependent apoptosis [54]. As a result, mitochondrial fission and fusion have two possible outcomes and functions depending on the physiological setting. Apoptosis is an early stage of liver injury. Cr(VI) can cause apoptosis via the mitochondria [55]. Cytochrome c (Cyt C), positioned at the interface of IMM and OMM, can be effectively retained within the mitochondria through the actions of OPA1 and Mfns. Drp1 aggregates on the OMM under different pressures and interacts with Bax to facilitate the translocation of pro-apoptotic proteins such as Cyt C and AIF into the cytoplasm, as well as activating the critical downstream effector protease caspase-3 in the apoptotic pathway, triggering apoptosis mediated by the mitochondria [56,57,58]. Previous studies revealed that Cr(VI) induces mitochondrial dynamics disorders and abnormal expression of apoptosis-related factors, as evidenced by the increasing mRNA and protein expression of mitochondrial-mediated apoptosis, such as Cyt C, Bax, and caspase-3, as well as Bcl-2 and the Bcl-2/Bax ratio in liver tissues and cardiomyocytes [47,59]; this is consistent with our results. Therefore, our study provides compelling evidence that Cr(VI) causes mitochondrial pathway apoptosis by inhibiting the AMPK/PGC-1a signaling pathway in duck livers.

## 4. Materials and Methods

### 4.1. Animal Treatment

Forty-eight Tianfu meat ducks (Nanchang Miao Wang Industrial Co., Ltd. (Nanchang, China)) were given a basic diet to adapt for 7 days, and a 12 h light/dark cycle was maintained. Then, all of the ducks were randomized into 4 groups (12 ducks per group). The final dietary contents of Cr(VI) in the 4 groups were as follows: 0 (control), low dose of K_2_Cr_2_O_7_ (9.28 mg/kg LCr), medium dose of K_2_Cr_2_O_7_ (46.4 mg/kg MCr), and high dose of K_2_Cr_2_O_7_ (232 mg/kg HCr) respectively. The dosage of hexavalent chromium was referenced to previous experimental results [42]. The source of Cr(VI) was 99.8% pure potassium dichromate (K_2_Cr_2_O_7_) of analytical grade from Heng da Chemical of Tianjin, China. The ducks were given an overdose intravenous injection of sodium pentobarbital (50 mg/kg) on the 49th day following a 12 h fast. Then, their livers were removed, and the samples were prepared and preserved according to the experimental needs. The feed formula references can be found in Table 1.

### 4.2. Histological and Ultrastructural Analyses

H&E staining was carried out in accordance with a previous technique [60]. In short, we cleaned the fresh liver tissue, cut it to a suitable size, and fixed it in 4% paraformaldehyde solution. Then, after a series of steps, such as gradient dehydration, wax immersion, embedding, etc., the embedded liver tissue was cut into 5 μM thick sections and, finally, stained with hematoxylin–eosin. Morphological changes in liver the tissue samples were evaluated using light microscopy (BX-FM: Olympus Corp, Tokyo, Japan).

### 4.3. Transmission Electron Microscopy

The procedure for sample preparation was followed [61]. Liver tissue segments (<1 mm^3^) were fixed with 2.5% glutaraldehyde and phosphate buffer for 2 h or longer, rinsed with 0.1 M phosphate-buffered saline (PBS), fixed with 1% citric acid, and then rinsed again with 0.1 M PBS. After dehydration, soaking, embedding, polymerization, trimming, and slicing, the segments were stained with uranyl acetate and lead citrate. We used transmission electron microscopy (TEM) (Hitachi H-7650, Tokyo, Japan) to examine the ultrastructure of the liver samples.

### 4.4. Determination of the Liver Function

The activities of alkaline phosphatase (AKP) (Cat No. A059-2-2), aspartate aminotransferase (AST) (Cat No. C010-2-1), and alanine aminotransferase (ALT) (Cat No. C009-2-1) in serum were detected according to the experimental method provided by the Nanjing Jiancheng Biological Kit to evaluate the degree of liver injury.

### 4.5. Detection of Antioxidant Index

The 10% liver tissue homogenate was obtained through homogenization in cold phosphate buffer (pH 7.4) and then centrifuged at 2500 rpm for 10 min at 4 °C, and then the protein concentration of each sample was determined by the BCA method (Solarbio: PC0020). The contents of glutathione (GSH) (Cat No. A006-2-1), malondialdehyde (MDA) (Cat No. A003-1-2), and hydrogen peroxide (H_2_O_2_) (Cat No. A064-1-1), along with the activities of catalase (CAT) (Cat No. A007-1-1), superoxide dismutase (SOD) (Cat No. A001-4-1), total superoxide dismutase (T-SOD) (Cat No. A001-3-2), and glutathione peroxidase (GSH-Px) (Cat No. A005-1-2), were each measured in accordance with the instructions on the respective kits.

### 4.6. Detection of Mitochondrial Function Index

The 10% liver tissue homogenate was obtained through homogenization in cold phosphate buffer (pH 7.4) and then centrifuged at 2500 rpm for 10 min at 4 °C, and then the protein concentration of each sample was determined by the BCA method. Adenosine triphosphate (ATP) (Cat No. A095-1-1), citrate synthase (CS) (Cat No. A108-1-1), and malate dehydrogenase (MDH) (Cat No. A021-2-1) in the liver were determined in accordance with the guidelines of the respective kits (Nanjing Jiancheng Bioengineering Institute, Nanjing, China).

### 4.7. Determination of Apoptosis by TUNEL Staining

According to the manufacturer’s instructions, the transferase-mediated deoxyuridine triphosphate–biotin nick stop labeling (TUNEL) kit from Keygen (Nanjing, China) was used to identify apoptotic cells in the liver. First, paraffin sections were dewaxed and infiltrated with 1% Triton X-100. After adding 3% H_2_O_2_–methanol, protease K was added at 37 °C for 10 min. The sections were then washed three times with PBS at 37 °C and stained with 4,6-diamino-2-phenylindole (DAPI). Finally, fluorescent microscope was used to find and record the stained apoptotic cells (Olympus BX41; Nikon, Tokyo, Japan).

### 4.8. Quantitative Real-Time PCR Analysis

Real-time quantitative polymerase chain reaction (RT-qPCR) was used the same precise experimental methodology [62]. Using the Trizol reagent (Takara, Japan), total RNA was extracted from liver samples and subsequently reverse-transcribed into reverse-transcription products (cDNA) for RT-qPCR. Also, the outcomes were analyzed using Applied Biosystems analysis software version 1.3. The primer sequence login numbers are shown in Table 2.

### 4.9. Western Blot Analysis

The total protein content of the liver samples was determined using the BCA protein detection kit (Solarbio, Beijing, China); the method was same as described by Wang et al. [63]. The primary antibodies of PGC-1α (1:5000) (Cat No.66369-1-lg), GAPDH (1:5000) (Cat No.10494-1-AP) were procured from Proteinates. Optic atrophy factor 1 (OPA1) (1:500) (Cat No. sc-393296), mitofusin 1 (Mfn1) (1:500) (Cat No. sc-166644), mitofusin 2 (Mfn2) (1:500) (Cat No. sc-515647), dynamin-related protein 1 (Drp-1) (1:500) (Cat No. sc-271543), human fission factor-1 (Fis1) (1:500) (Cat No. sc-376447), and mitochondrial fission factor (MFF) (1:500) (Cat No. sc-398617) were procured from Santa Cruz., while AMPK (1:200) (Cat No. WL02254), p-AMPK (1:200) (Cat No. WL05103), Bcl-2 (1:200) (Cat No. WL01556), Bax (1:200) (Cat No. WL01637), Cyt C (1:200) (Cat No.WL02410), and cleaved caspase-3 (1:200) (Cat No.WL02117) antibodies were procured from Wanleibio. The Bio-Rad ChemiDoc Touch imager (Bio-Rad ChemiDoc Touch CA, USA) picked up the signal. Finally, the grey cost of the corresponding protein was analyzed by way of ImageJ software (ImageJ, RRID:SCR_003070 https://imagej.net/ij/).

### 4.10. Statistical Analysis

Intergroup differences were analyzed by one-way ANOVA using SPSS version 25.0, drawn using GraphPad Prism 9.0. Significant differences between groups are denoted by lowercase letters, and extremely significant differences are denoted by uppercase letters.

## 5. Conclusions

In summary, Cr(VI) exposure can induce oxidative stress, mitochondrial dynamics disorders, and mitochondrial pathway apoptosis by inhibiting the AMPK-PGC-1α signaling pathway in duck livers.

## Figures and Tables

**Table 1:** Composition and nutrient levels in the basal diet for the ducks.

Composition of Diet	Content (%)		Nutrient Levels		Level
Ingredient	0~3 Weeks	After 3 Weeks	Index	0~3 Weeks	After 3 Weeks
Soybean meal	18.00	20.00	Ca (%)	0.800	2.77
Corn	59.99	44.00	DE (MJ·kg^−1^)	11.93	11.44
Wheat bran	11.00	14.40	Crude protein (%)	18.03	17.63
Rice bran	—	11.00	Met + Cys (%)	0.600	0.650
Cottonseed meal	5.00	—	Total phosphorous (%)	0.670	0.700
Bone meal	1.58	5.80	Lys (%)	0.850	0.970
Fish meal	3.00	2.00	Available phosphorus (%)	0.350	0.400
Salt	0.370	0.300			
Met	0.060	0.100			
CaHPO_4_	—	1.40			
Premix *	1.00	1.00			
Total	100	100			

* Each kilogram of premix contained the following: V_D3_ 400 IU, V_A_ 2500 IU, V_B_ 1215.0 μg, V_K3_ 0.5 mg, V_E_ 10.0 mg, riboflavin 4.0 mg, thiamine 4.0 mg, nicotinic acid 55.0 mg, pantothenic acid 11.0 mg, biotin 0.08 mg, pyridoxine 2.5 mg, folic acid 1.0 mg, choline 1300.0 mg, Se 0.20 mg, Fe 80.0 mg, Cu 10.0 mg, Zn 60.0 mg, Mn 50.0 mg.

**Table 2:** Gene primer sequences and their GenBank accession numbers.

Gene	Accession Number	Primer Sequences (5′ to 3′)
*Mfn1*	XM_027464235.2	Forward: TAAAGTCTCCTCTGCCATGACC Reverse: ACGGTTTACAAGTGAAGTCCA
*Mfn2*	XM_027443332.2	Forward: CTGGCATTGATGTAACCAC Reverse: CAAAGAAAATTCGATCCCCT
*OPA1*	XM_027463877	Forward: ACAATGCCTTAGAAGATCGGTCA Reverse: CTTTTATCAGACAGGGGTCCAC
*MFF*	XM_038183985.1	Forward: TAAAATGCCACGGTTCCAGT Reverse: TCACAGTGCAATCCTTAGTCG
*Bcl-2*	XM_005026830.4	Forward: GGAGGGCTCTGAAAGAAAAGG Reverse: TATGATGCGATGGCACGACTG
*Bax*	KY788660.1	Forward: GCCATCAAGGCTCTGTTCTCGC Reverse: TCAAGGCGCTGTCCTCGCCATTTTCCA
*Caspase-3*	XM_005030494.1	Forward: CGGACTGTCATCTCGTTCAGGCAC Reverse: GTCCTTCATCGCCATGGCTTAGC
*Cyt C*	XM_027447873.1	Forward: ACAAAGGAGATGGCAATGCA Reverse: CACCCCACATATGAGCAACG
*β-Actin*	EF667345.1	Forward: ATGTCGCCCTGGATTTCG Reverse: CACAGGACTCCATACCCAAGAAT

## Data Availability

The datasets analyzed during the current study are available from the corresponding author upon reasonable request.
